# Constructing Multifunctional Composite Paper Coated with Polypyrrole@Lignocellulosic Slurry with Humidity Sensing, Conductivity, Antibacterial, and Photothermal Properties

**DOI:** 10.3390/polym17070898

**Published:** 2025-03-27

**Authors:** Qingrun Ni, Yating Wang, Shoujuan Wang, Magdi E. Gibril, Fangong Kong

**Affiliations:** State Key Laboratory of Green Papermaking and Resource Recycling, Key Laboratory of Pulp & Paper Science and Technology of Shandong Province/Ministry of Education, Faculty of Light Industry, Qilu University of Technology, Shandong Academy of Sciences, Jinan 250353, China; nqr15650519180@163.com (Q.N.); wangyating0028@163.com (Y.W.); magdi.gibril@gmail.com (M.E.G.)

**Keywords:** lignocellulosic slurry, polypyrrole, multifunctional paper, humidity sensing, conductivity, antibacterial activity, photothermal response

## Abstract

A multifunctional paper-based composite of paper coated with a polypyrrole@lignocellulosic slurry (PPy@LS) and carboxymethyl cellulose (CMC) was developed. PPy@LS was prepared via the polymerization of pyrrole onto a lignocellulosic slurry derived from hemp stalks prepared using deep eutectic solvents. The PPy@LS slurry was mixed with the required amount of CMC and vacuum-filtered onto filter paper to fabricate the composite (PPy@LS/CMC). The resulting composite paper exhibited excellent multifunctional properties, including electrical conductivity, photothermal conversion, and antibacterial properties. These properties are stable against external environments, such as water and abrasion, due to the addition of CMC. The electrical conductivity of PPy@LS/CMC varied in the dry (1.6 × 10^−4^ S/cm) and wet (4.8 × 10^−6^ S/cm) states, suggesting its potential application in humidity sensing. Notably, the PPy@LS/CMC paper achieved significant photothermal activity under light irradiation, as demonstrated by the measured surface temperature exceeding 80 °C in 10 min. Moreover, the composite paper exhibited > 99.9% antibacterial activity against Escherichia coli (Gram-negative) and Staphylococcus aureus (Gram-positive). The combination of the inherent characteristics of filter paper along with the photothermal property of PPy enable the PPy@LS/CMC composite appropriate for solar interfacial evaporation application. These multifunctional composite papers with innovative combinations of properties have great potential for applications in smart packaging, humidity sensing, biomedicine, and solar-driven water purifications.

## 1. Introduction

The development of multifunctional paper-based materials with adjustable properties has recently attracted significant interest because of their possible use in many different disciplines, including smart packaging, sensing, photothermal conversion, and biomedicals [1]. Based on their sustainability, low cost, and tunable design, the global market for multifunctional paper, which includes smart packaging, sensing, photothermal conversion, and biomedical applications, is expected to grow from USD 182.5 to 416.5 billion by 2030 [2]. The many benefits of paper-based materials include low cost, environmental friendliness, abundant hydroxyl groups for great modifiability, porous structure, high specific surface area, separation performance, and simplicity of recovery and recycling [3]. The structural features of multifunctional paper-based materials make them particularly appropriate for membrane separation technology, thereby improving their practical use in many fields. Nevertheless, unmodified paper-based materials have low wet strength and poor water resistance, which limits their practical use [4]. Chemical modification, coating, and other treatments are required to overcome these constraints [5]. Coating paper with conductive polymers, including polypyrrole (PPy), which has attracted a lot of interest because of its sustainability, tunability, and adaptability, seems to be one interesting direction. Polypyrrole has been extensively applied in sensors, electromagnetic shielding, and energy storage systems owing to its outstanding electrochemical characteristics, environmental stability, and tunable conductivity [6]. In addition to its straightforward synthesis methods, PPy exhibits superior electrical conductivity properties compared to other common conductive polymers, making it a wise choice for constructing conductive paper-based surfaces [7]. However, adding PPy to paper substrates presents challenges, including limited durability, poor adherence, and insufficient interaction between PPy and cellulose fibers. Researchers have investigated several approaches to solve these problems using the interface between PPy and paper substrates. One method tackles the inherent limitations of polypyrrole by merging it with other materials to create hybrid composites that incorporate a paper substrate [8,9]. This approach was developed to maximize the interaction and compatibility between PPy and paper, which may improve the stability, adhesion, and general performance of the coatings [10]. Previous studies have effectively produced paper-based composites using this approach. Many studies have focused on preparing multifunctional paper-based flexible, conductive, and high-performance composites by integrating PPy with materials such as polymers, lignin, cellulose nanofibers (CNFs), silver nanoparticles (Ag), 4-butylbenzenesulfonate, reduced graphene oxide (rGO), and MXene [11,12]. Among these materials, lignin, a naturally occurring polymer present in plant cell walls, has become increasingly appealing as a component for improving the quality of paper-based products [13]. It is a by-product of the pulp and paper sector and has fluorescent, antibacterial, and natural antioxidant properties. Its unique chemical composition makes it an ideal candidate for diverse and environmentally friendly composites. Recently, flexible and conductive polypyrrole (PPy) functional paper has been produced from lignin [14,15]. For PPy, lignin was used as a dispersant and dopant, and the mechanical strength and electrically stable PPy paper-based composite material were demonstrated. Similarly, Dong [16] developed a high-mechanical electrode with excellent capacitive performance by decorating a sodium lignosulphonate/polypyrrole interpenetrating network onto the LCNFs. Lignin shows natural antioxidant, antibacterial, and fluorescence properties, but its inconsistent properties and limited processability are ascribed to the severe lignin extraction techniques used, which may remove important functional groups from lignin, thus reducing its properties [17,18]. Deep eutectic solvents (DESs) have recently become intriguing green solvents for lignin extraction [19]. DES extraction systems based on acids provide various advantages over the traditional approaches. The high acidity of DES promotes simultaneous lignin extraction and cellulose defibrillation, thus producing a cohesive slurry of defibrillated cellulose and regenerated lignin [20]. Thus, we hypothesized that the lignocellulosic slurry prepared by DES would act as a stabilizer, delivering a composite with highly distributed lignin and nanofibrillated cellulose coated with PPy, which could be used for paper coating. Thus, we expect that the coupling of PPy and lignocellulosic slurry on paper substrates will offer an opportunity to generate multifunctional paper with desired properties. These characteristics include enhanced photothermal conversion, mechanical strength, electrical conductivity, and antibacterial capabilities. The applications of such multifunctional papers include flexible electronics, smart packaging, and environmental sensing among other areas. To improve the flexibility and durability of multifunctional paper, modest amounts of carboxymethyl cellulose (CMC) must be added as a binder or cohesive agent. However, to the best of our knowledge, the utilization of the lignocellulosic slurry prepared using DES for the production of multifunctional composite paper has not been reported. Therefore, this study aimed to develop and fabricate multifunctional composite paper using a lignocellulosic slurry coated with PPy, evaluate its physicochemical properties, and explore its potential applications.

In this study, a simple method was proposed to build a totally bio-based multifunctional paper using a composite of PPy@LS. Three stages yielded a composite functional paper with several characteristics, including photothermal, antimicrobial, and conductivity: (1) Using deep eutectic solvents as a pretreatment for agro-waste (hemp stalks), (2) PPy@LS made by in situ polymerization of PPy, and (3) the necessary quantity of CMC added to the composite slurry and vacuum-filtered onto filter paper as a substrate. Although lignin and nanofibrillated cellulose improved the mechanical properties, PPy improved the conductivity and photothermal properties. Furthermore, the introduction of lignin and PPy significantly affected the antibacterial activity at a rate of more than 99.9%. composite paper had a notable impact on antibacterial activity, with a rate of more than 99.9%. CMC improved the durability, flexibility, and water absorption of the composite paper and closed the holes and crevices of the paper substrate. Under solar irradiation, the composite paper also exhibited remarkable photothermal conversion, with the surface temperature reaching a maximum of 80 °C in 10 min. Among the various benefits of the recently produced multifunctional paper are its simplicity of preparation, whole biological base, biodegradability, and multifarious functionality.

## 2. Materials and Methods

### 2.1. Materials

Hemp stalk (Hs) was provided by Heilongjiang Tianzhicao Bio-New Material, Heilongjiang, China). Choline chloride ((C_5_H_14_ClNO) analytical grade (purity: 98%, Mw: 139.62)) and oxalic acid dihydrate ((H_2_C_2_O_4_·2H_2_O) analytical grade (purity: 99.5%, Mw: 126.07)) were obtained from the Shanghai, China McLean Biotechnology Co. Sodium carboxymethyl cellulose (CMC) (Mw ~250,000, degree of substitution ~0.9, and viscosity: 300–800 mPa·s) was purchased from Shanghai, China Reagent Co., Ltd. Hydrochloric acid (HCl, 37%) was purchased from Yantai Far East Fine Chemical Co. (Yantai, China), whereas potassium persulfate ((K_2_S_2_O_8_) ACS reagent, ≥99.0%) and pyrrole (reagent grade 98%) were purchased from Shanghai, China McLean Biotechnology Co.

### 2.2. Preparation of Regenerated Lignin-Cellulose Slurry (LS) from Hemp Stalks

**Figure 1:**
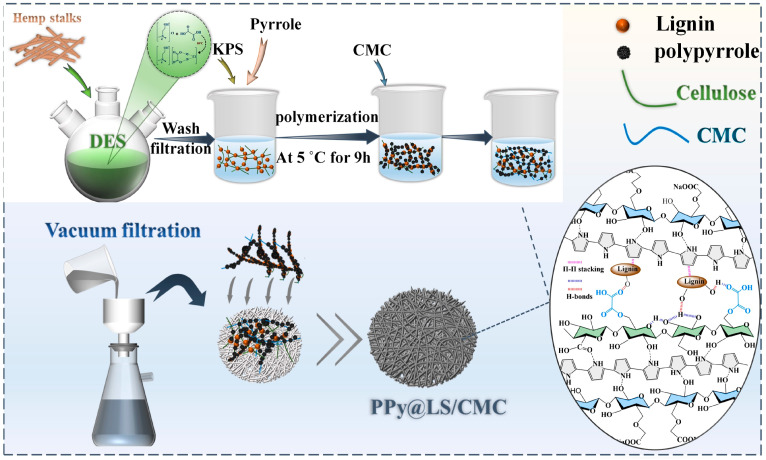
Schematic of the synthesis of PPy@LS/CMC.

The LS (lignocellulosic slurry) derived from hemp stalks was prepared using methods described in the literature and according to scheme in Figure 1 [21]. Initially, DES was prepared by combining choline chloride (ChCl) and oxalic acid (OA) in a 1:1 molar ratio, heated to 80 °C until a transparent DES liquid was formed. The hemp stalk was crushed and sieved through a 60-mesh screen to generate a powder, which was subsequently treated with the DES solution in a reactor at a mass ratio of 1:15 to produce a lignocellulosic slurry. The mixture was heated at 110 °C for 4 h under continuous stirring. A highly viscous brown solution was produced owing to the dissolution of the lignin and hemicellulose. Subsequently, deionized (DI) water was added to the mixture at a volume ratio of five times the volume of the DES. The mixture was continuously stirred for 1 h and allowed to precipitate for 6 h. The LS was isolated from the DES water system by filtration using a 0.22 μm filter membrane. The filtrate was subjected to multiple washings (5–7 times) and vacuum filtration to remove any residual DES until the pH reached 7. This LS slurry typically contains approximately 12% solid material by weight.

### 2.3. Preparation of PPy@LS and PPy@LS/CMC Paper-Based Composites

According to scheme in Figure 1, LS (0.1 g) was dispersed in 100 mL of water and the pH was adjusted to 2 using hydrochloric acid. CMC (0.5 g) was stirred at 50 °C until dissolution and cooled to room temperature. Under ice bath conditions, 5 mL of polypyrrole was added to the solution while maintaining vigorous agitation, and the required amount of water-soluble potassium persulfate (KPS) was gradually introduced as an initiator. The mixture gradually turned black, indicating the polymerization of polypyrrole. The mixture was maintained under consistent conditions for 9 h to ensure complete polymerization. Subsequently, 40 g of the mixture was vacuum-filtered onto filter paper to obtain the PPy@LS/CMC-0.5 sample. Finally, the prepared PPy@LS and PPy@LS/CMC paper-based composites were dried at 70 °C for 12 h. These steps were repeated to alter the amount of CMC to 0, 1, and 1.5 g to obtain the other three samples: PPy@LS/CMC-0, PPy@LS/CMC-1, and PPy@LS/CMC-1.5.

### 2.4. Characterization

The chemical structures of the samples were analyzed using Fourier transform infrared spectroscopy (FTIR) (Bruker VERTEX 70, Ettlingen, Germany) in the wavelength range of 370–7500 cm^−1^ with a spectral resolution of 0.9 cm^−1^. An X-ray diffraction (XRD) instrument (Bruker D8 ADVANCE, Ettlingen, Germany) was used to analyze the crystalline structure of the samples using a copper target with a wavelength of 1.5418 Å in the reflectance mode. Thermal analysis was conducted on the samples using an STA449 F3 instrument from Germany, with temperatures ranging from 25 to 600 °C at a heating rate of 10 °C/min in a nitrogen environment. All samples were coated with a gold layer using ion-sputtering coater (MC1000—Hitachi High-Tech, Tokyo, Japan) before testing. The surface morphologies of the paper and composite samples were examined using a scanning electron microscope (SEM, Hitachi Regulus 8220, Tokyo, Japan), and an ImageJ analyzer WiKi (1.14 version) was used to calculate the particle size. X-ray photoelectron spectroscopy (XPS, ESCALa-b220i-XL, West Sussex, UK) was used to analyze the surface chemistry of the samples, and the electrical properties of the samples were measured at room temperature under ambient atmosphere with a probe station on a Keithley 4200 SCS semiconductor parameter analyzer. The temperature change in the sample at a solar intensity (1 kW m^−2^) simulated by a xenon lamp (CME-SL500, Iwata, Japan) for 0 to 17 min was measured using an infrared thermal imager (FLIR ONE PRO, Wilsonville, OR, USA). To evaluate the antibacterial activity of the samples, the antibacterial properties of the membrane-forming solution against Escherichia coli (Gram-negative) and Staphylococcus aureus (Gram-positive) were evaluated. The bacteriostatic zone size of the bacteriostatic zone was used to determine the bacteriostatic capability. therefore, DPPH (2,2-diphenyl-1-pyridinium) free radical scavenging test to study the free-radical scavenging ability of the films, thereby measuring their antioxidant properties. The static contact angle (CA) of the coated surface was determined using an automatic contact angle measuring device (OCA50, Dataphysics, Filderstadt, Germany).

## 3. Results and Discussion

### 3.1. Characterization of PPy@LS

**Figure 2:**
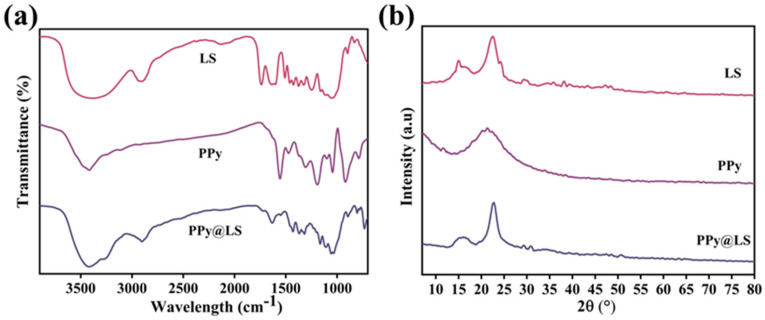
(**a**) FTIR spectra and (**b**) XRD patterns of PPy, LS, and PPy@LS.

As illustrated in Figure 2a, the PPy spectrum exhibits peaks at 1560 cm^−1^, 1306 cm^−1^, 1183 cm^−1^, 1029 cm^−1^, 918 cm^−1^, and 778 cm^−1^ corresponding to C=C (valence vibration in PPy ring), C-H, (in-plane stretching), C-N (stretching), C-N (deformation), N-H (out-of-plane vibration), and N-H (out-of-plane vibration), respectively. This indicates the synthesis of the PPy polymer [22]. The FTIR spectrum of LS exhibited all the fundamental characteristic peaks associated with lignocellulosic materials. For instance, a strong broad O-H stretching frequency is evident near 3390 cm^−1^. A pronounced C-H vibration was observed at 2910 cm^−1^. The bands observed between 1623 cm^−1^ and 1417 cm^−1^ correspond to the aromatic skeleton vibrations in lignin. The peaks observed at 1321–1371 cm^−1^ correspond to the C-H bending of cellulose and lignin [23]. The weak peak at 1213 cm^−1^ corresponds to the C-O stretching of the phenolic hydroxyl groups in lignin, whereas the weak peak at 1053 cm^−1^ is associated with the C-O stretching of cellulose and lignin. In addition to these peaks, the PPy@LS composite spectrum showed the main characteristic peaks of PPy at around 1550 cm^−1^ and 1450 cm^−1^ corresponding to C-C and C-N stretching in the pyrrole ring, respectively [24]. This confirmed the PPy polymerization and synthesis of the PPy@LS composite.

Figure 2b presents the X-ray diffraction (XRD) patterns of PPy@LS and PPy. PPy prepared by polymerization exhibited a broad XRD peak with an amorphous characteristic at 2θ = 23°, which was attributed to scattering from PPy chains at the interplanar spacing. In contrast, three peaks were observed for PPy@LS at 2θ = 14.5°, 16.5°, and 22.5°, corresponding to the cellulose type-I structure and lignin [25]. However, compared to the LS diffraction, the strength of these peaks decreased in LS. The PPy@LS peak at 22.5° is broader than the LS peaks owing to the addition of the PPy layer. The estimated degree of crystallinity of PPy@LS (48.3%) was lower than that of LS (55.6%) and higher than that of PPy (40%). This was due to the amorphous structure of PPy. These findings imply that the PPy@LS composite was successfully constructed.

**Figure 3:**
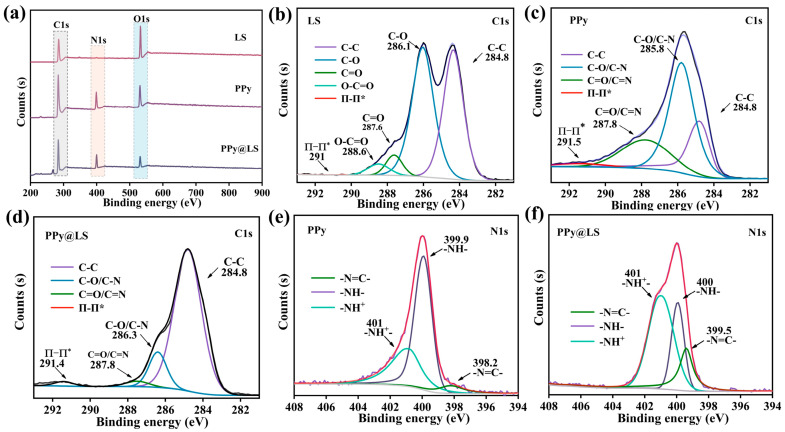
(**a**) Full-scan XPS C 1s spectra of PPy, LS, and PPy@LS; (**b**) XPS C 1s spectra of LS; (**c**) XPS C 1s spectra of PPy; (**d**) XPS C 1s spectra of PPy@LS; (**e**) N 1 s spectra of PPy; and (**f**) N 1 s spectra of PPy@LS.

The chemical bonding and elemental composition of LS before and after polymerization with PPy were characterized using XPS. As illustrated in Figure 3a, the XPS survey scan of PPy@LS exhibited C1s, N1s, and O1s peaks at 285, 400.1, and 532 eV, respectively. Conversely, the XPS survey of LS demonstrated C1s and O1s peaks at 285 and 532 eV, respectively. Compared to LS, PPy@LS displayed a distinct N1s peak at 400.1 eV, indicating the presence of PPy. To confirm this observation, C1s deconvolution was performed, and the obtained results are shown in Figure 3b–d. The C1s spectrum of PPy@LS was deconvoluted into five subpeaks at 284.8, 285.7, 286.5, 287.9, and 288.9 eV, corresponding to C–C/C–H, C–O, C–N, C=O, and O=C–O, respectively (Figure 3d). The deconvoluted C1s spectrum of PPy@LS exhibited signals corresponding to C associated with C–O/C–N (286.4 eV), C=O/C=N (287.8 eV), C–NH (288.6 eV), and π–π interactions (291.4 eV) [26]. Similarly, the deconvoluted N1s spectra of PPy and PPy@LS were examined. The N1s detailed spectrum of PPy@LS exhibits several variations. In comparison to the N1s spectrum of PPy (Figure 3e), the N1s spectrum of PPy@LS in Figure 3f shows a shift from a symmetric peak to an asymmetric peak [27]. In addition, a shoulder peak at 401 eV was observed, indicating PPy aggregation on the lignin moieties. These findings are in agreement with the literature [28] and suggest strong hydrogen bonding between the OH groups on cellulose and lignin with the NH of PPy, which elucidates the robust adherence of PPy on LS. This is evidenced by the absence of PPy detachment after numerous washing cycles of PPy@LS for 2 h.

**Figure 4:**
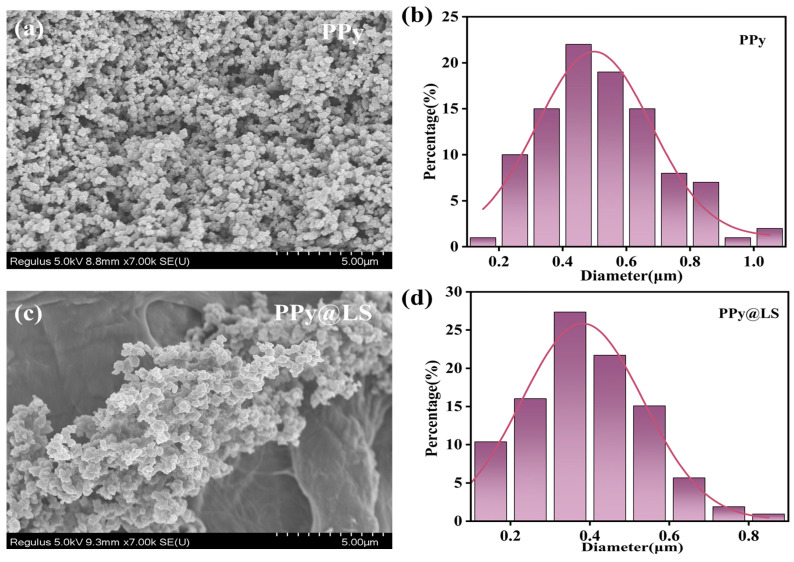
SEM images of (**a**) PPy and (**c**) PPy@LS and histograms of PPy (**b**) and PPy@LS (**d**).

Figure 4 shows the histograms and SEM images of PPy and PPy@LS, respectively. Well-defined particles shapes were observed in both Figure 4a,c. As shown, PPy exhibits dense particles with a narrow range of sizes, and an average particle size in the particles in range of 390–610 nm is observed in the SEM image of PPy (Figure 4b). Figure 4c shows that PPy@LS exhibited a narrow range of sizes, with an average particle size in the range of 210–490 nm (Figure 4d). The homogeneous distribution of nanoparticles in PPy@LS indicates strong interactions between lignin and PPy, resulting in a compact structure [22]. However, the average size of the PPy particles in PPy@LS slightly decreased because lignin prevented the agglomeration of PPy nanoparticles, that is, lignin acted as a dispersing agent.

**Figure 5:**
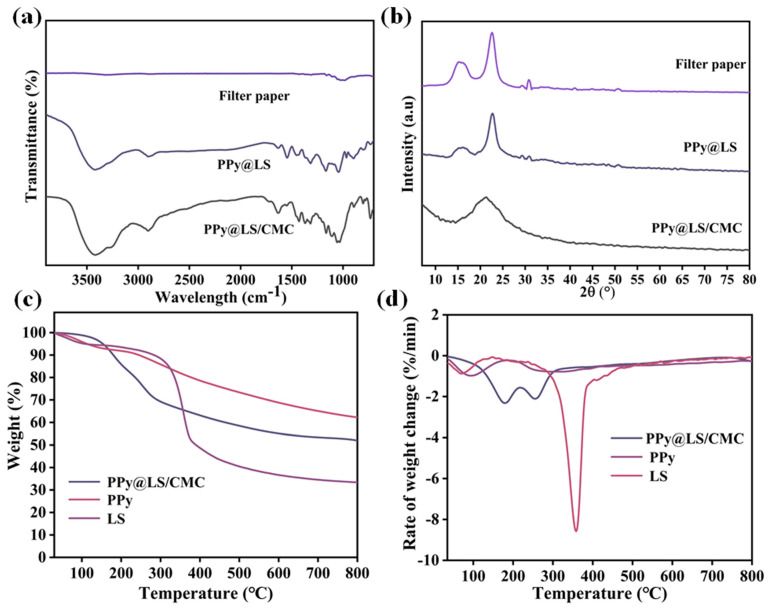
(**a**) FTIR curves of filter paper, PPy@LS/CMC-0, and PPy@LS/CMC; (**b**) XRD patterns of filter paper, PPy@LS/CMC-0, and PPy@LS/CMC; (**c**) TGA, and (**d**) DTG of LS, PPy, and PPy@LS/CMC.

Figure 5a shows The FTIR spectra of the filter paper (FP), filter paper coated with PPy@LS, and filter paper coated with PPy@LS/CMC composites. The FTIR spectrum of the filter paper exhibited typical characteristic peaks for cellulose materials at 1430 cm^−1^ (CH_2_ scissoring), 1374 cm^−1^ (C–H deformation), 1316 cm^−1^ (CH_2_ wagging), 1160 cm^−1^ (symmetrical stretching vibration of C–O–C), and 1060–1015 cm^−1^ (C–O stretching vibration in cellulose) [29]. Compared to the FP spectrum, the spectra of FP coated with PPy@LS and PPy@LS/CMC showed new peaks, confirming the addition of PPy@LS and PPy@LS/CMC to FP, respectively. For instance, PPy@LS exhibited additional peaks at 1542 and 1635 cm^−1^, which correspond to the characteristic peaks of PPy and LS, respectively [30,31]. However, after the addition of CMC, the PPy@LS/CMC spectrum revealed two new peaks at 1722 cm^−1^ and 1421 cm^−1^ corresponding to the stretching of the COO—group in the carboxyl group of CMC [32]. The PPy@LS/CMC spectra showed overlapping absorption bands for both components, except for the C-C and C-N bending modes of the pyrrole ring. The presence of tiny peaks at 1557 and 1470 cm^−1^ and the decreased bending of the LS OH groups suggest that PPy was loaded at LS. The peak at 1530 for pure polypyrrole was shifted higher. These findings confirm that the presence of LS affects the skeletal vibrations of the delocalized π-electrons in the polypyrrole chain. In addition, the peak of N-H wag at 770 cm^−1^ in the PPy spectrum disappeared in the spectrum of PPy@LS/CMC due to the chemical bonding between -N-H in the pyrrole and the -OH functional group of LS, as stated by Song et al. [33].

Filter paper well-defined crystalline peaks are seen in Figure 5b. The most notable peaks were observed at two less intense positions at 2θ = 14.9°and 16.6°and a sharp peak at 2θ = 22.8, which correspond to the (110), (110), and (002) crystalline planes of natural cellulose, respectively [34]. For the composites of FP coated with PPy@LS and PPy@LS/CMC, the main characteristic peaks of cellulosic paper were still present with some changes. In the case of FP coated with PPy@LS, after the addition of PPy@LS to the paper, the diffraction peaks were 14.5°, 16.5°, and 22.5°, corresponding to the cellulose type I structure; however, the peak at 22.5° became broad because of the characteristic peaks of lignin and PPy [35,36]. However, after CMC of the mixture, the FP coated with PPy@LS@CMC exhibited a clear crystal diffraction peak at 2θ = 21.17°. This indicates the success of the composite material fabrication [37].

Figure 5c,d show the thermal performance curves (TGA and DTG) of LS, PPy, CMC, and PPy@LS/CMC, respectively. As shown in Figure 5c, the first stage of weightlessness occurred between 90 °C and 120 °C, mainly due to the release of water. PPy has good thermal stability; in the case of the PPy heat map, owing to the degradation of PPy, the weight loss is only 30% in the temperature range of 30–800 °C. In contrast, LS showed a similar thermal behavior with a weight loss of 80% in the same temperature range. This occurred because of the breakdown of cellulose and lignin components. Compared to LS and PPy, PPy@LS/CMC showed the same thermal characteristics; however, it exhibited thermal degradation with a weight loss of 40%, which is higher than that of LS and lower than that of PPy. This phenomenon is attributed to the formation of a protective layer on the cellulose surface by PPy and lignin, which exhibit higher thermal stabilities [38].

**Figure 6:**
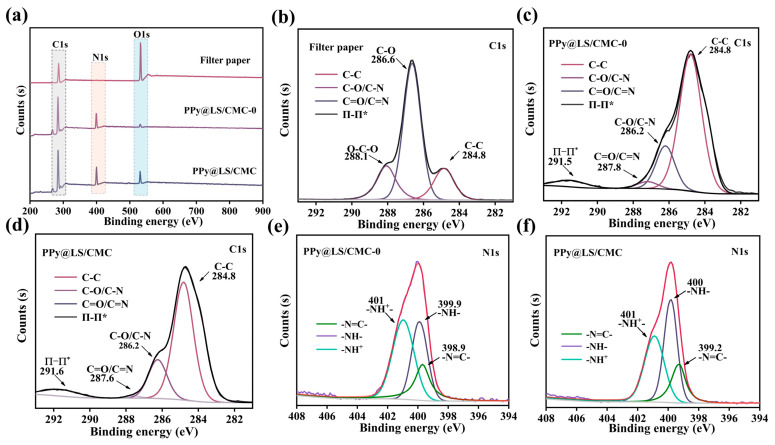
(**a**) Full-scan XPS C 1s spectra of filter paper, PPy@LS/CMC-0, and PPy@LS/CMC, (**b**) XPS C 1s spectra of filter paper, (**c**) XPS C 1s spectra of PPy@LS/CMC-0, (**d**) XPS C 1s spectra of PPy@LS/CMC, (**e**) N 1 s spectra of PPy@LS/CMC-0, and (**f**) N 1 s spectra of PPy@LS/CMC.

The surface chemical constituents of FP, PPy@LS/CMC (0), and PPy@LS/CMC were analyzed using X-ray photoelectron spectroscopy (XPS). As shown in Figure 6a, the XPS spectrum of FP exhibits peaks at 285.1 and 532.5 eV, which are assigned to the C1s and O1s photoelectrons, respectively. However, PPy@LS/CMC (0) showed C1s, O1s, and N1s, the presence of N1s is confirming the presence of PPy. Compared with PPy@LS/CMC(0), the XPS curve of FP coated with PPy@LS/CMC showed C1s, O1s, N1s, and traces of Na. The presence of Na and the changes in the C1s and O1s intensities indicate the introduction of CMC onto the coated paper. In addition, resolution-enhanced carbon spectra of FP, PPy@LS/CMC (0), and PPy@LS/CMC were obtained using the deconvolution method, as shown in Figure 6b, c, and d, respectively. The XPS C1s spectra of FP, PPy@LS/CMC (0), and PPy@LS/CMC (Figure 6d) reveal that FP exhibits only three primary components: C-C, C-O, and O-C-O bonds at 284.8, 285.8, and 287.8 eV, respectively [39]. Compared to FP, PPy@LS/CMC(0) exhibited three different carbon atom types: C–C (284.8), C-O/C-N (285.8), and C=O/C=N (287.8); however, the intensities and shapes of these peaks were different. Similarly, the C1s spectrum of PPy@LS/CMC showed four different carbon atom types: C-C (284.8), C-O/C-N (285.8), C=O/C=N (287.3), and O-C=O/N-C=O (288.6), indicating the successful preparation of composite paper materials [40]. In addition, the deconvoluted N1s spectra of PPy@LS/CMC (0) and PPy@LS/CMC were examined (Figure 6e,f). The N 1s spectra of PPy@LS/CMC(0) and PPy@LS/CMC(0) show some changes. In comparison to the N1s of PPy@LS/CMC(0) (Figure 6e), the N1s spectra of PPy@LS/CMC at (401), in Figure 6f, displayed that the intensity of N1s at 401 was decreased due to the formation of a symmetric peak to an asymmetric peak of PPy [27]. In addition, a shoulder peak at 401 eV was observed, indicating the interaction of PPy@LS with the CMC moieties. These findings are in agreement with the literature [28] and suggest strong hydrogen bonding between the OH of CMC and the NH of PPy, which elucidates the robust adherence between PPy@LS and CMC.

**Figure 7:**
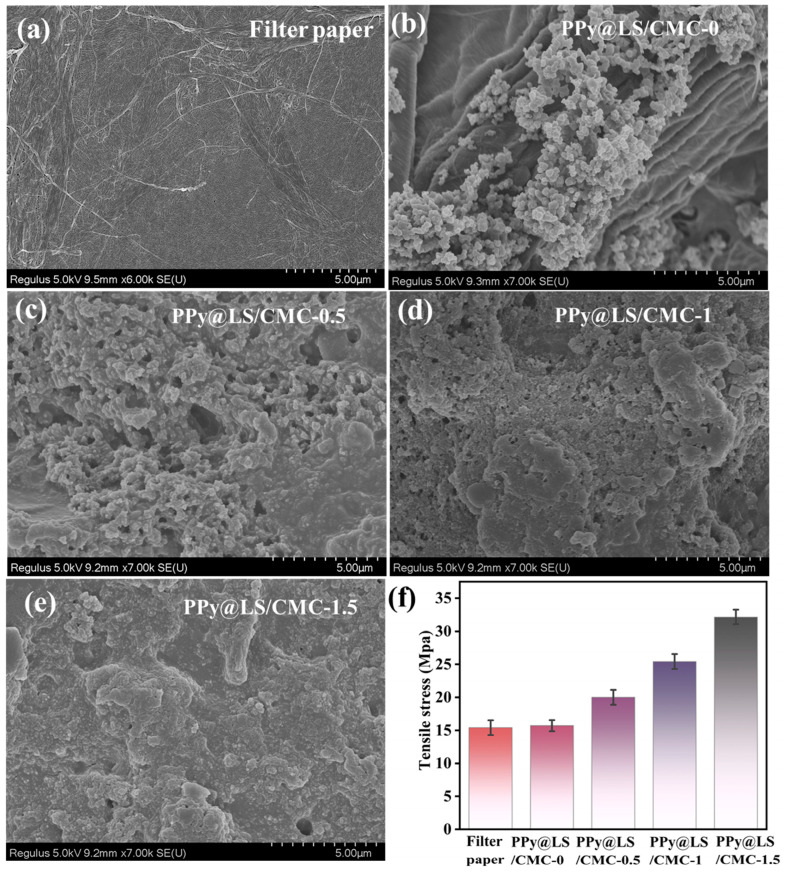
(**a**) SEM image of filter paper, (**b**) SEM image of PPy@LS/CMC-0, (**c**) SEM image of PPy@LS/CMC-0.5, (**d**) SEM image of PPy@LS/CMC-1, and (**e**) SEM image of PPy@LS/CMC-1.5, (**f**) tensile properties of the sample.

The SEM images show the surface microstructures of the different materials (Figure 7). The image clearly shows that the surface of the filter paper has a dense, smooth arrangement of fibers (Figure 7a), and PPy covered the surface with a denser structure, smaller size, uniform particles, and spherical PPy particles (Figure 7b). The SEM micrographs of the PPy@LS/CMC composite samples depicted in Figure 7c–e reveal randomly distributed dense particles that appear as spots of varying sizes on their surfaces. These spots indicate the presence of PPy and CMC and exhibit variations in roughness across all samples. This finding suggests the segregation of PPy@LS within the CMC, which may be validated by the interaction and complexation of PPy@LS/CMC with FP.

The effects of PPy@LS and PPy@LS/CMC on the tensile properties of FP were also investigated. As illustrated in Figure 7f, FP had the lowest tensile strength of 15 MPa compared to the other samples. Applying the PPy@LS coating to the FP increased the tensile strength to 17 MPa. This enhancement was attributed to the strong adhesion of PPy, lignin, and cellulose nanofibrils to FP. Furthermore, incorporating varying amounts of CMC into the composite system led to a substantial increase in the tensile strength, ranging from 20 to 30 MPa. The tensile strength was positively correlated with the amount of CMC added to the system. This enhancement in tensile strength can be attributed to two factors. The PPy@LS coating likely formed a cohesive layer on the FP surface, reinforcing the fiber network and improving the overall mechanical properties. The addition of CMC further strengthened the composite structure by forming additional hydrogen bonds between the cellulose fibers of FP and the coating components (PPy@LS) [41]. As the CMC content increased, it may have also acted as a binding agent, improving the interfacial adhesion between the paper fibers and conductive coating, resulting in a more robust and mechanically resilient material.

**Figure 8:**
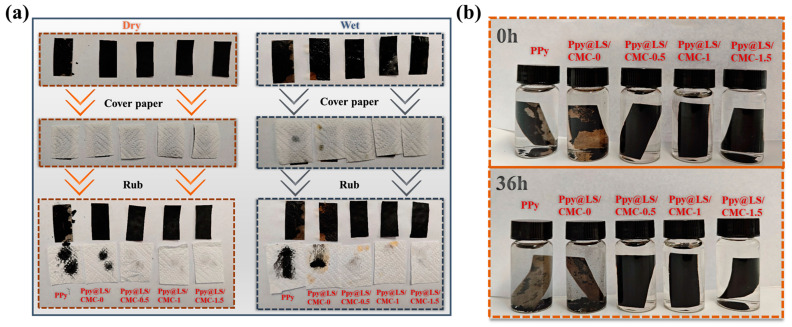
(**a**) Adhesion degree of surface material of different samples, and (**b**) water stability of the sample.

Figure 8a shows a comparison of the adhesion ability between the surface materials and paper under dry and wet conditions for the composite materials. Kitchen paper was used to wipe the surfaces of the materials under both dry and wet conditions. The image clearly shows that the material with only PPy has an obvious shedding phenomenon, and after adding LS, although there is also a shedding phenomenon, it is obviously improved, indicating that LS increases the adhesion between PPy and FP. This phenomenon was significantly improved after the addition of CMC, which ensured that the surface material of the filter paper was firmly attached to the filter paper in both the dry and wet states. As can be seen in Figure 8b, after 36 h of different materials were placed in water, the surface material of the material without CMC fell off, while the material with CMC maintained its original shape. This is because the good interaction between CMC and PPy@LS formed a protective layer on the surface, resulting in strong water stability.

Based on the outstanding results of the FP coated with PPy@LS/CMC, it was selected for further characterization. This composite material combines the conductive properties of PPy with the structural advantages of the LS structure and properties, with the CMC acting as a binding agent. The presence of CMC not only improves the adhesion between the components but also potentially influences the overall conductivity and other properties of the composites. Therefore, composite paper with different CMC fractions was used to investigate its effect on the photothermal, conductivity, and antibacterial properties.

### 3.2. Electrical Conductivity of PPy@LS@CMC

**Figure 9:**
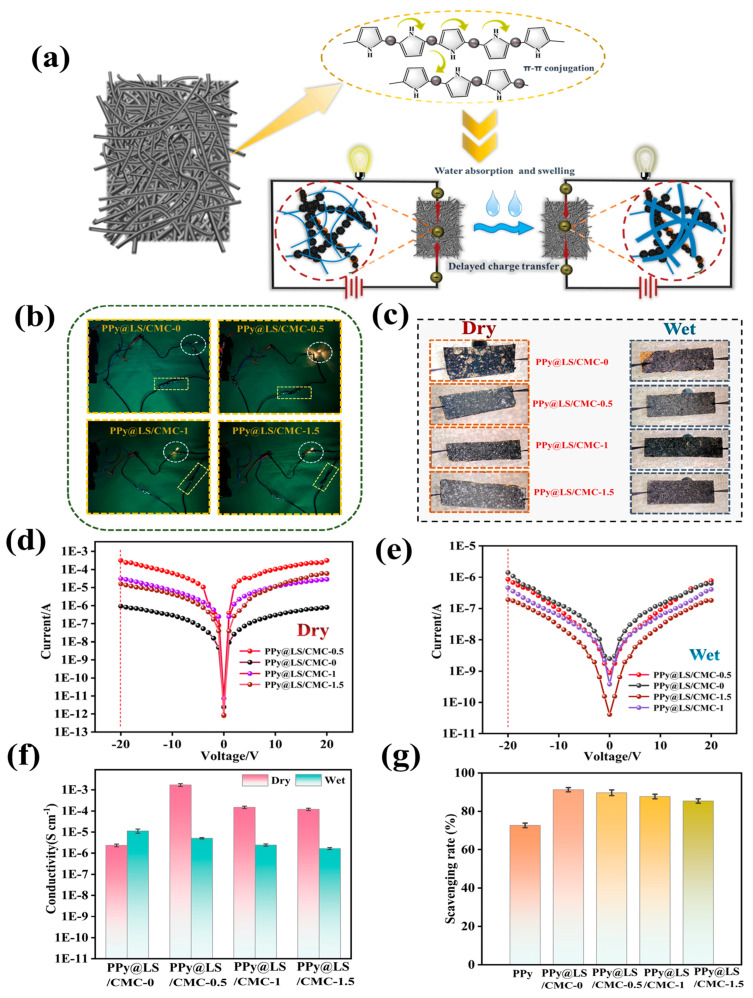
(**a**) PPy@LS/CMC electrical conduction mechanism diagram, (**b**) sample conductivity test, (**c**) sample surface morphology, (**d**,**e**) (I–V) curves of the sample under wet and dry conditions, (**f**) conductivity of the sample under wet and dry conditions, and (**g**) oxidation resistance of the sample.

The electrical conductivities of the PPy@LS/CMC composites are shown in Figure 9a. PPy exhibits a conjugated structure characterized by alternating carbon-carbon single and double bonds. A double bond comprises σ and π electrons [42,43]. The σ electrons are immobile and form covalent bonds with the carbon atoms. In contrast, the two π electrons in the conjugated double bond are not confined to a single carbon atom; they can translocate from one carbon atom to another, thus extending across the molecular chain. Consequently, the conductivity of polypyrrole involves the movement of positively charged carriers and electrons along the polymer chain, as well as the movement of these carriers between the chains [44,45]. In the presence of an electric field, the electrons constituting the π bonds can migrate along the molecular chain. Therefore, PPy imparts electrical conductivity to the material [46]. Because of the CMC coating on the PPy@LS/CMC surface, when CMC comes in contact with water, it hydrates to form a larger insulating layer covering the conductive PPy surface, resulting in a reduction in its electrical conductivity [47]. This phenomenon allowed PPy@LS/CMC to respond to changes in conductivity under wet and dry conditions, rendering it potentially applicable in the field of intelligent humidity-responsive materials [48].

**Figure 10:**
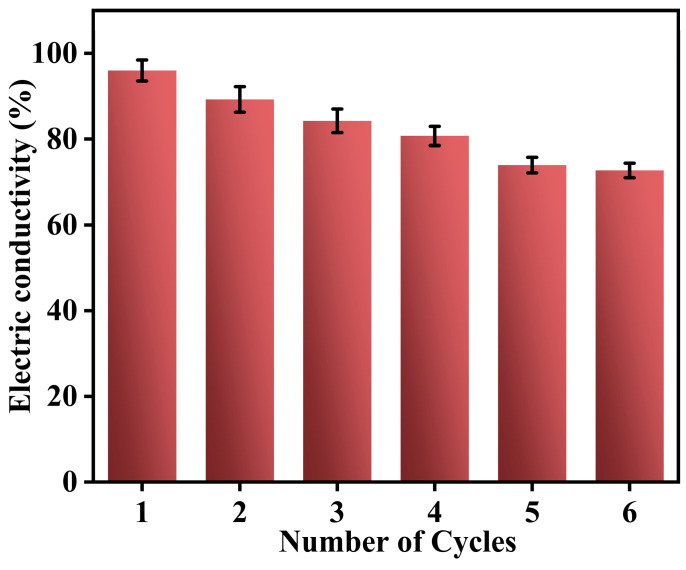
The conductivity stability of the PPy@LS/CMC after 6 cycles.

As shown in Figure 9a, when the sample was incorporated into a complete circuit, the LED lamp emitted bright light, with PPy@LS/CMC-0.5 exhibiting the highest luminosity among the samples. This result can be explained by examining the microscopic morphology of the sample during the electrical conductivity tests (Figure 9b). As the CMC content increased, the surface of the sample became denser and more compact because of the augmentation of the insulating layer on the surface. However, the electrical conductivity of the material without CMC was inferior to that of the sample containing CMC because, in the absence of the binding effect of CMC, PPy on the surface of the sample was prone to detachment, resulting in a larger gap between the surface and the conductive material, thereby reducing its electrical conductivity. Figure 9d,e demonstrate that the conductivity of the composite material was confirmed through current density and voltage (I-V) measurements, and the filter paper attached to PPy transitioned from an insulator to a semiconductor between an insulator and a conductor [49]. The electrical conductivities of the samples in the dry state corresponded to their microscopic morphologies. In the wet state, the electrical conductivity of PPy@LS/CMC-0 increased because of the introduction of conductive water, whereas the electrical conductivities of the other samples decreased because of the increase in CMC on the surface. Figure 9f shows a map of the conductivity of the sample in both wet and dry states, further substantiating this observation. This variation suggests potential applications in the field of intelligent, humidity-responsive materials. One of the primary limitations of PPy is its poor performance stability, particularly its electrical conductivity. PPy interacts with oxygen in ambient air, resulting in aging and a reduced conductivity [50]. The antioxidant activities of the different samples were evaluated using a DPPH free radical scavenging assay (Figure 9g) to assess the antioxidant capacity. PPy exhibited a DPPH clearance rate of 72%. After the addition of LS, the free radical clearance rate of PPy@LS/CMC-0 reached 91% owing to the presence of lignin, and it slightly decreased after the addition of CMC. The free radical clearance rate of PPy@LS/CMC-0.5 was 89%, showing a slight decrease. This decrease can be attributed to the encapsulation that CMC encapsulates the LS on the surface by CMC. With an increase in CMC, the free radical clearance rate of the sample continued to decrease but remained higher than that of the PPy. The results indicate that the introduction of LS significantly improved the oxidation resistance of the material and enhanced the service life of the conductive material. The PPy@LS/CMC exhibited almost consistent conductivity over six consecutive cycles, as shown in Figure 10, indicating its excellent conductivity stability under variable weather conditions (humidity) and power density.

### 3.3. Photothermal Property of PPy@LS/CMC

**Figure 11:**
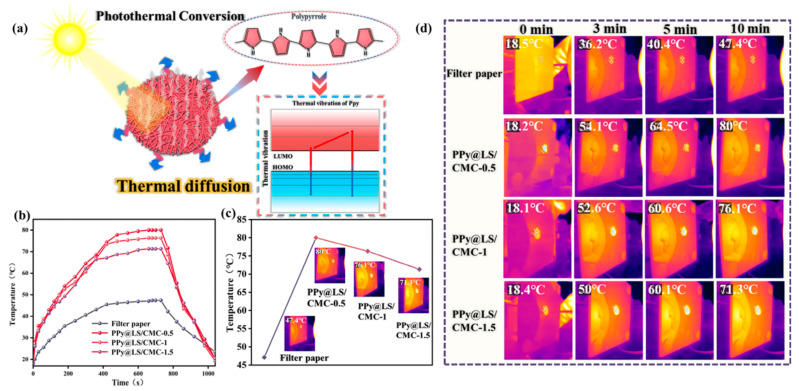
(**a**) PPy@LS/CMC photothermal mechanism diagram, (**b**) the sample temperature changes with time under xenon lamp irradiation, (**c**) maximum surface temperature for different samples, and (**d**) infrared thermal image of the sample under xenon lamp irradiation.

As demonstrated in Figure 11a, the photothermal conversion performance of PPy@LS/CMC was primarily attributed to the presence of the PPy coating on the surface of the material and abundant electron retention in the PPy conjugate structure [51]. Electromagnetic radiation can induce excitation from the π orbital of the highest occupied molecular orbital (HOMO) to the π* orbital of the lowest unoccupied molecular orbital (LUMO) [52]. Following this transition, the electrons in the LUMO return to the ground state through electron–phonon coupling, and the thermal energy from lattice vibration is released during this relaxation process, resulting in an increase in the macroscopic temperature of PPy@LS/CMC, which enhances its photothermal properties. An additional factor contributing to this phenomenon is the presence of lignin in the LS on the surface [53]. Despite its low concentration, studies have demonstrated that the adjacent benzene rings of lignin are nearly coplanar, forming extensive conjugated π bonds and numerous π–π stacks [54]. This configuration enhances electron delocalization, reduces the energy required for electron transition, and confers photothermal conversion properties to lignin [55]. For this purpose, a xenon lamp with a light intensity of 1 kW m^−2^ was used as the visible-light analog source, and the photothermal conversion effect of each material was investigated (Figure 11b–d). The composite paper exhibited rapid heating to >50 °C within 3 min, and the maximum surface temperature was attained within 10 min. The filter paper alone reached a maximum surface temperature of approximately 47 °C, whereas the composite material reached a surface temperature of up to 80 °C (Figure 11c,d). With an increase in the CMC content, the maximum surface temperature of the composite material gradually decreased, which was attributed to the reduction in the PPy content on the surface.

### 3.4. Contact Angle and Antibacterial Activity

**Figure 12:**
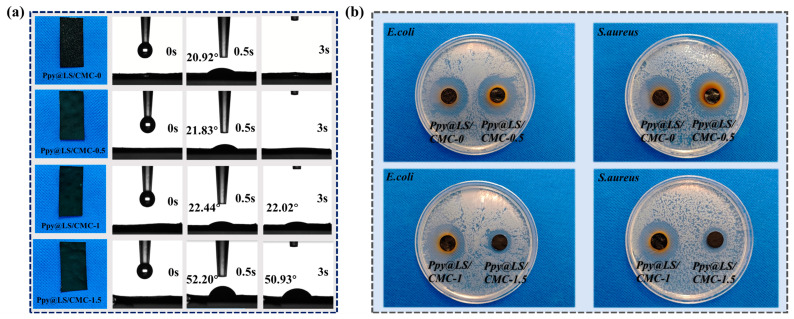
(**a**) Water contact angle of the samples and (**b**) antibacterial properties of the samples.

Figure 12a presents the test results for the contact angles of different materials. It has been well established that filter paper exhibits good water permeability; therefore, the contact angle for FP could not be determined as a drop of water soon penetrated. After the addition of PPy@LS, the FP exhibited a water contact angle of 20.92°, whereas, after the addition of PPy@LS/CMC, the water contact angle increased slightly (21.83°). This enhancement is attributed to the hydrophobic nature of PPy and lignin, which could also be related to the morphology of the surface [56]. This result is in agreement with data reported by Carlos et al. [28] However, with an increase in the CMC content, PPy@LS/CMC-1 and PPy@LS/CMC-1.5 showed an enhancement in water contact angles. This phenomenon can be attributed to the increased density of the filter paper surface with a higher CMC content, which affects water transmission. This could also be attributed to the interaction between lignin and CMC as a result of the drying process. In addition, carboxylation of CMC alters its hydrophilicity, impeding water penetration [32].

Escherichia coli (*E. coli*) and Staphylococcus aureus (*S. aureus*) were used as bacterial strains, and the antibacterial effects of various samples were evaluated using the AGAR diffusion method. As shown in Figure 12b, all samples exhibited distinct inhibition zones. PPy@LS/CMC-0 and PPy@LS/CMC-0.5 demonstrated nearly identical inhibitory zones that were marginally larger than those of PPy@LS/CMC-1, whereas PPy@LS/CMC-0.5 exhibited the smallest inhibitory zones. These results indicate that PPy demonstrates excellent antibacterial properties [57]. As the CMC content increased, PPy was deposited on the sample surface, resulting in a decrease in antibacterial activity. The superior photothermal properties and water permeability suggest that PPy@LS/CMC has potential applications in solar interface evaporators. The prolonged use of bio-based evaporators in aquatic environments can readily promote bacterial growth, leading to a reduction in their service life [58]. Consequently, the excellent antibacterial properties of PPy@LS/CMC enhanced its durability in various aqueous environments, rendering it more suitable for application in this field and as a smart-packaging material.

## 4. Conclusions

This study presents a simple approach for synthesizing bio-based multifunctional paper using a lignocellulosic slurry (LS) and a polypyrrole (PPy) composite on a paper substrate with carboxymethyl cellulose (CMC) as the adhering agent. The PPy@LS/CMC composite paper was prepared through three processes: pre-treatment of hemp stalks with deep eutectic solvents, in situ polymerization of PPy, and the addition of CMC. The results revealed that the composite papers exhibited excellent mechanical strength, photothermal properties, conductivity, and antibacterial activity. It is obviously that the PPy@LS/CMC functional paper has potential applications across several domains due to its unique and essential properties. Its conductivity facilitates its use in flexible electronics and smart packaging, and its humidity-sensing properties render it appropriate for environmental monitoring systems. The antibacterial and photothermal properties of the composite paper can be used for sterilizing instruments, wound dressings, and solar interfacial evaporation applications. This study presents a processing concept for fabricating paper-based composites by combining green chemistry and agro-waste valorization, which could serve as a platform for developing a new class of multifunctional papers.

## Data Availability

The original contributions presented in the study are included in the article, further inquiries can be directed to the corresponding authors.

## Abbreviations

The following abbreviations are used in this manuscript:

PPy	polypyrrole
LS	lignocellulosic slurry
PPy@LS	polypyrrole@ lignocellulosic slurry composite
PPy@LS/CMC	polypyrrole@ lignocellulosic slurry composite adhered with carboxymethylcellulose
FP	filter paper
CMC	carboxymethyl cellulose
